# Fallopian tubal histogenesis of ovarian endometriosis—A study of folate receptor-alpha expression

**DOI:** 10.3389/fmed.2023.1138690

**Published:** 2023-03-02

**Authors:** Yiying Wang, Qiyan Li, Ruijiao Zhao, Jerry Y. Wang, Yan Wang, Wanrun Lin, Zeng Yuan, Jing Zhang, Oluwole Fadare, Yue Wang, Wenxin Zheng

**Affiliations:** ^1^Department of Obstetrics and Gynecology, Henan Provincial People’s Hospital, Zhengzhou University People’s Hospital, Henan University People’s Hospital, Zhengzhou, China; ^2^Department of Obstetrics and Gynecology, Henan Provincial People’s Hospital, Henan University People’s Hospital, Zhengzhou, China; ^3^Department of Pathology, Henan Provincial People’s Hospital, Zhengzhou University People’s Hospital, Henan University People’s Hospital, Zhengzhou, China; ^4^Department of Pathology, University of Texas Southwestern Medical Center, Dallas, TX, United States; ^5^Department of Obstetrics and Gynecology, Qilu Hospital, Shandong University, Shandong, China; ^6^Department of Biological Sciences, University at Albany, SUNY, Albany, NY, United States; ^7^Department of Pathology, University of California San Diego, San Diego, CA, United States; ^8^Department of Obstetrics and Gynecology, University of Texas Southwestern Medical Center, Dallas, TX, United States; ^9^Department of Pathology, Harold C Simmons Comprehensive Cancer Center at University of Texas Southwestern Medical Center, Dallas, TX, United States

**Keywords:** ovarian endometriosis, fallopian tube, tubal origin of endometriosis, biomarkers of endometriosis, FRA

## Abstract

**Background:**

Ovary is a common organ site involved by endometriosis. We previously found that fallopian tube may contribute to the histogenesis of ovarian endometriosis. The finding was novel and requires further studies. We addressed this issue by examining a differentially expressed gene folate receptor alpha (*FOLR1*) and its protein (FRA) in this study.

**Results:**

A total of 144 tissue samples were studied. These included 32-paired tubal-endometrial-ovarian endometriosis samples (*n* = 96), 18 samples of ovarian endometriosis without corresponding fallopian tube or endometrium, and 30 ovarian tissue samples with ovarian surface epithelia but without endometriosis. Multiple comparisons among groups of ovarian endometriosis, normal fallopian tube and benign endometrium were performed. *FOLR1* was highly expressed in the epithelia of fallopian tube and ovarian endometriosis, with paired endometrial samples showing a significantly lower level of expression. Similar differential studies for FRA protein were performed through Western blot and immunohistochemistry (IHC). The expression of folate receptor alpha at both mRNA and protein levels in the tissues (fallopian tube or ovarian endometriosis vs. the endometrium) were significantly different (*p* < 0.001). All ovarian surface mesothelial epithelia showed negative expression of FRA by IHC.

**Conclusion:**

The results further support that the fallopian tube may contribute to the development of ovarian endometriosis. Understanding the tubal contribution to ovarian endometriosis should ultimately contribute to ongoing investigative efforts aimed at identifying alternative ways to prevent and treat endometriosis. High level of FRA expression in the fallopian tube and endometriosis might be considered as potential tissue sites for targeted therapy.

## Background

Endometriosis, one of the most common gynecologic disorders, is a bewildering and debilitating disease that affects millions of women in the world. Endometriosis, defined as the presence of endometrial glands and stroma in extrauterine sites. Endometriosis can be divided into several types based on the anatomic locations. Peritoneal endometriosis is typically located in the pelvic surface of peritoneum or on the ovary. Ovarian endometrioma or endometriotic cyst describes the lesion in the ovary when endometriosis forming a cystic structure. Deep infiltrating endometriosis mostly represents a solid mass comprised of endometriotic tissue with local adipose and fibromuscular tissue within pelvic structures between the rectum and the vagina. The ovarian endometriosis is the most common. Clinical symptoms of endometriosis are numerous and may include dysmenorrhea, dyspareunia, menorrhagia, dyschezia, pelvic and abdominal pain, infertility, and symptoms from gastrointestinal tract.

Pathogenesis of endometriosis remains unclear. The most popular hypothesis is the retrograde menstruation theory, which was originally proposed by Sampson (1) in 1927. Although it is popular, it remains controversial for a variety of reasons. Retrograde menstruation is thought to occur in up to 90% of reproductive age women (2), but only 6–10% of these women have clinical symptoms related to endometriosis (2). Additionally, the theory falls flat to explain the presence of endometriosis outside the peritoneal cavity. The coelomic metaplasia hypothesis proposed by Meyer (3) states that endometriosis may originate from mesothelial cells through a metaplastic process, although how the metaplasia happens is ambiguous. Initial endometriosis, which our group described in 2005 (4), represents a spectrum of the earliest morphologically identifiable changes of endometriosis within the ovary. At that time, we assumed that the morphologic transitions from ovarian surface epithelia (OSE) or ovarian epithelial inclusions (OEI) to initial endometriosis lesions represented a metaplastic process from OSE as discussed in 2005 (4). However, nowadays we realize that the majority of epithelia attached on the ovarian surface and OEIs are derived from the fallopian tubal epithelia. This was found when we studied the origin of OEIs and the low-grade serous carcinoma from the fallopian tube epithelia vs. ovarian surface mesothelia in 2011 (5). We are far from understanding the molecular mechanism of pathogenesis of endometriosis. More recently, Koninckx et al. proposed a novel wholistic genetic/epigenetic theory of the pathogenesis of endometriosis (6). Based on this theory, the set of genetic and epigenetic incidents transmitted at birth could explain many aspects of the endometriosis including hereditary, predisposition, immunology, and placentation. To develop cystic ovarian endometriosis, Koninckx et al. believed that a series of additional transmissible genetic/epigenetic incidents are required to occur in a cellular level, including stem or stem-like cells (6). Interestingly, this genetic/epigenetic theory is compatible with the most, if not all, the observations made on endometriosis including subtle microscopic lesions we described as “initial endometriosis” (4).

The fallopian tube, particularly tubal fimbriated end, has recently caught great attention in studies of adnexal pathology and the origin of ovarian serous cancers. This is mainly because most investigators understand that the majority ovarian serous cancers are actually derived from the fallopian tube, not the ovary as was historically believed (7–9). Similarly, in our study of the cellular origins of OEIs, we found that the majority OEIs (also called endosalpingiosis) display a phenotype suggesting they are derived directly from tubal epithelial cells rather than from ovarian surface mesothelial cells (5, 10). Indeed, microscopic examination of resected ovarian and tubal tissues frequently shows various stages of the following sequence: tubal epithelia mostly from fimbriated end are frequently adherent on the ovarian surface, accompanied by invagination of morphologically similar epithelia onto ovarian surface, then invaginate into the cortex forming OEIs or endosalpingiosis (5, 7). From those findings, we believe that the formation of ovarian initial endometriosis occurs through the conversion of tubal-like epithelia within the ovarian cortex into endometriosis-like tissue, rather than the conversion of ovarian surface mesothelial cells into endometriosis-like tissue. The potential for tubal mucosal epithelia to convert into endometriosis-like tissue is also supported by the phenomenon of “endometrialization” of the proximal end of the fallopian tube after a tubal ligation procedure (11, 12). Based in part on the aforementioned morphological findings, we first proposed the hypothesis that tubal epithelia contribute to the formation of ovarian endometriosis in 2014 and 2015 (10, 13).

The biomarker FRA (folate receptor alpha), the product of the *FOLR1* gene, has previously been shown to be differentially expressed between the fallopian tube and the endometrium (14, 15). We have also noticed that *FOLR1* was differentially expressed in our previous gene expression array study (10). We did not study *FOLR1* and FRA in detail at that time mainly because the antibody against FRA was not available. It is interesting to know if FRA is expressed in the ovarian endometriosis. Understanding the level of FRA expression in ovarian endometriosis may help understanding cellular lineage of endometriosis and potential target therapy. In this study, we aimed to investigate FRA expression in paired tissue samples of human ovarian endometriosis and normal tubal and endometrial tissue samples to further examine the cellular origin of the ovarian endometriosis.

## Results

### Multiple differentially expressed genes identified in the fallopian tube and the endometrium

A total of 4,114 and 3,451 genes were identified from the tubal and endometrial samples, respectively. The gene expression profiles of the paired tubal and endometrial samples were compared using a Volcano Plot. The threshold for a differentially expressed genes between the tubal and endometrium was set at ≥2.0-fold level. There were 1796 genes identified with more than two-fold differential expressions between the two tissue samples. All differentially expressed genes were further scrutinized and some representative genes were listed and studied previously (10).

Compared with the endometrial samples, the tubes showed a total of 911 upregulated genes. These included genes that were ≥50-fold (*n*=8), ≥20-fold (*n*=28), and ≥2-fold (*n* = 875) comparatively upregulated in the fallopian tube. There were no genes with more than 50-fold upregulation in the endometrial sample relative to the tubal tissue. *FOLR1* (gene bank accessing number NM_016729), which was not listed previously and represented another most differentially expressed gene, showed 55.6-fold increment in the tubal sample compared to the gene expression level at the endometrium (*p* = 0.012831689).

### FOLR1 gene detection in the fallopian tube, endometrium, and ovarian endometriosis by real-time PCR

**Figure 1:**
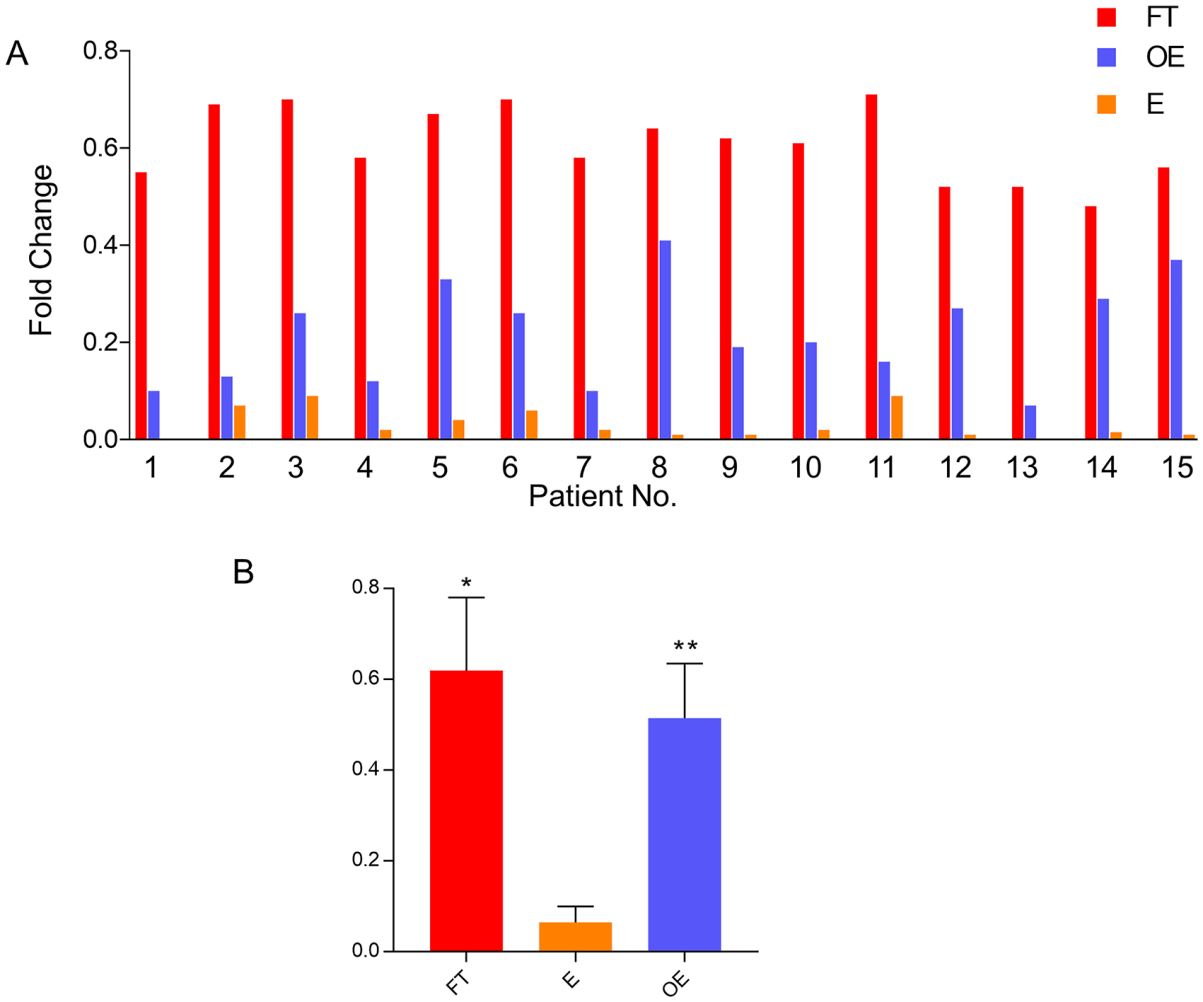
*FOLR1* gene expression level in paired tubal, endometrial, and ovarian endometriosis samples. Comparisons of mRNA expression levels in the fallopian tube, the endometrium, and the foci of endometriosis by quantitative PCR in 15 individual paired cases **(A)** and the pooled data **(B)**. *FOLR1* levels in fallopian tube (FT) and ovarian endometriosis (OE) were similar, but both were higher than the observed *FOLR1* level in the endometrium (E) [*p = 0.176 (FT vs. OE) and **p = 0.0018 (E vs. OE)]. FT: fallopian tube; E: endometrium; OE: ovarian endometriosis.

Real-time PCR was used to examine the level of *FOLR1* expression in 15 paired fresh tissue samples (total of 45 samples, including 15 each of the fallopian tube, endometrium, and ovarian endometriosis specimens). *FOLR1* was highly expressed in the fallopian tube compared with the paired endometrium, with fold increment ranging from 9 to 35 (average fold change = 18.25, *p* < 0.001). Similarly, the *FOLR1* gene was highly expressed in the samples of ovarian endometriosis compared with the paired endometrium, with fold increment ranging from 3 to 20 (average fold change = 11.18, *p* < 0.001). However, although there were some expression level differences between the fallopian tube and ovarian endometriosis samples, the difference did not reach statistical significance (*p* = 0.125). The gene expression level from the 15-paired cases, together with pooled data, is shown in Figure 1. Among these 15-paired samples, 12 pairs (from 1 to 12) were in the proliferative phase and 3 (13 to 15) were in secretory phase of the menstrual cycle (Figure 1). The gene expression levels of the two menstrual phases were compared and no significant differences (*p* > 0.10) in the organ-matched samples were found (data not shown).

### FRA protein expression in the fallopian tube, endometrium, and ovarian endometriosis by Western blot

**Figure 2:**
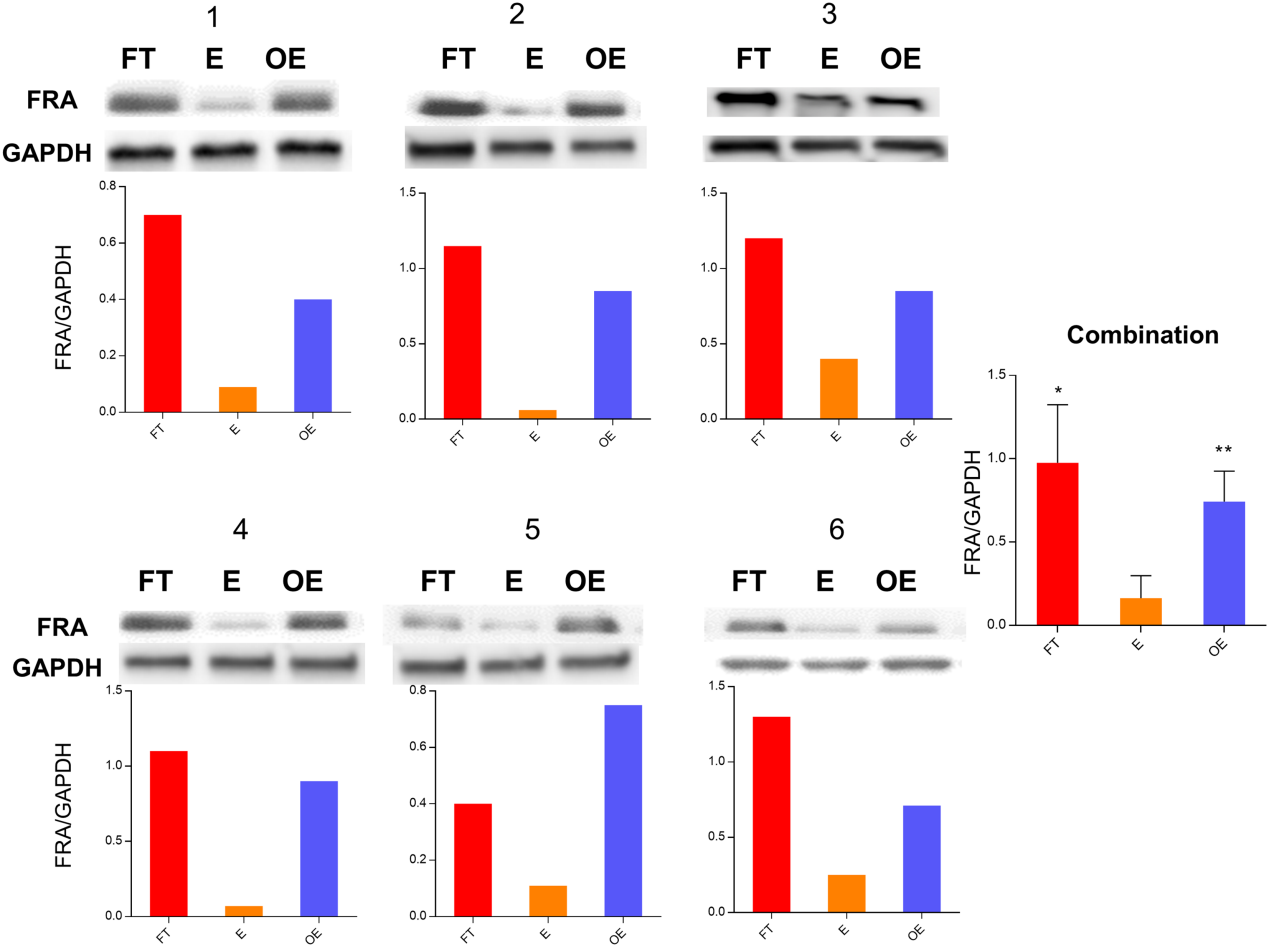
FRA protein expression level in paired tubal, endometrial, and ovarian endometriosis samples. Comparisons of protein expression levels of the fallopian tube (FT), the endometrium (E), and the foci of ovarian endometriosis (OE) by Western blot analyses. The figure shows the data from 6 paired samples with number labeled on the top of each panel. The FRA and GAPDH control bands are presented on the top, while the ratio of FRA/GAPDH is illustrated in the bottom for all 6 paired samples. The pooled data are summarized on the right side of the figure. Similar to the gene expression levels, FRA protein was significantly higher in the fallopian tube and the ovarian endometriosis samples than that in the endometrium [*p = 0.103 (FT vs. OE) and **p = 0.001 (E vs. OE)].

FRA protein level examination for the 15-paired samples was performed by Western blot. FRA protein expression was significantly higher in the fallopian tube and ovarian endometriosis samples than that in the endometrium, with an average fold of increment of 12.36 (*p* = 0.001) and 9.72 (*p* = 0.022), respectively. As expected, there were no significant differences between the fallopian tube and ovarian endometriosis samples (*p* > 0.10). These results were compatible with the findings from real-time PCR validation, indicating that *FOLR1* or FRA do not change significantly at the transcriptional and post-transcriptional levels. Western blots of 6 representative paired samples and pooled data of FRA protein expression are shown in Figure 2.

### FRA expression in tissue sections of the fallopian tube, endometrium, ovarian endometriosis and ovarian surface epithelia by immunohistochemistry

A total of 114 tissue samples were analyzed with anti-FRA antibodies by IHC. These tissue sections represented the 32 paired tissue sections of the fallopian tube, endometrium, and ovarian endometriosis and additional 18 samples of ovarian endometriosis. All 32 normal fallopian tube samples showed strong and diffuse FRA expression as expected. The observed staining pattern was mostly intense cytoplasmic, with some extending to the luminal border of tubal epithelia. The staining score ranged from 185 to 300 with an average of 228 for the tubes. There were minor differences in FRA staining between tubal secretory and ciliated cells, with the latter showing stronger staining intensity on the luminal border than the former. However, these staining differences were not statistically significant (data not shown). In addition, compared with the menstrual phases, proliferative versus secretory, we did not observe a significant difference of FRA staining pattern or intensity in the tubal and endometrial epithelia.

FRA expression in 32-paired and 18-non-paired ovarian endometriosis samples were also highly positive, with IHC scores ranging from 94 to 300 with an average of 160. The majority of the epithelial cells were positive for FRA stain. Among all the 50 cases with endometriosis, there were 38 cases showing staining intensity score 3, 8 score 2, and 4 score 1. There were no significant differences in FRA expression scores between the paired and non-paired endometriosis. FRA expression was negative in all Pax-8 negative OSE as well as in all Pax-8 negative OEIs (surface epithelium derived ovarian inclusions) from the 30 ovarian sections without endometriosis examined (data not shown). These findings suggest that mesothelial cell derived structures are negative for FRA, while tubal cell generated endosalpingiosis is positive for FRA.

In the eutopic endometrial tissue sections, FRA expression was largely absent, with IHC scores that ranged from 0 to 84 (average 42). Typically, only focal areas of endometrial glands were positive with weak intensity at the luminal apical borders of the epithelial cells. Among the 32 endometrial samples, 26 were in proliferative and 6 in secretory phase; Occasional weak staining was seen in samples of both proliferative and secretory endometria with no clearly discernible differences in staining frequency or intensity.

**Figure 3:**
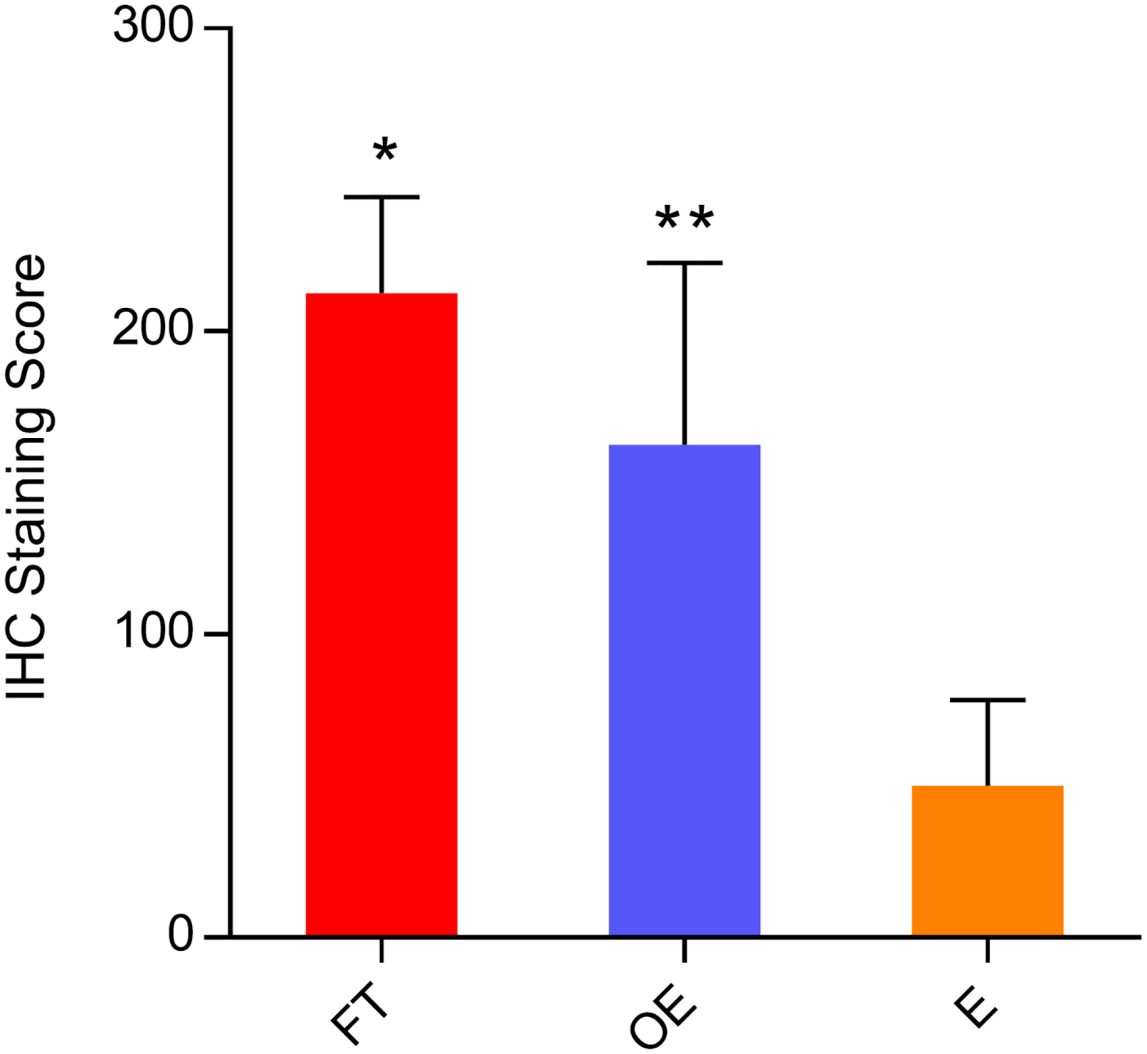
FRA protein expression levels by immunohistochemical scores in paired tubal, endometrial, and ovarian endometriosis samples. Comparisons of FRA protein expression levels in the fallopian tube, the endometrium, and the lesions of endometriosis by IHC scores in 32 paired and 18 endometriosis cases (pooled data): FRA expression was significantly higher in the fallopian tube and ovarian endometriosis than that in the endometrium [*p = 0.00051 (FT vs. E) and **p = 0.00174 (OE vs. E)]. The FRA expression level in the fallopian tube was also higher than the FRA level in the ovarian endometriosis, with the difference just attaining statistical significance (*p* = 0.05). FT: fallopian tube; E: endometrium; OE: ovarian endometriosis.

**Figure 4:**
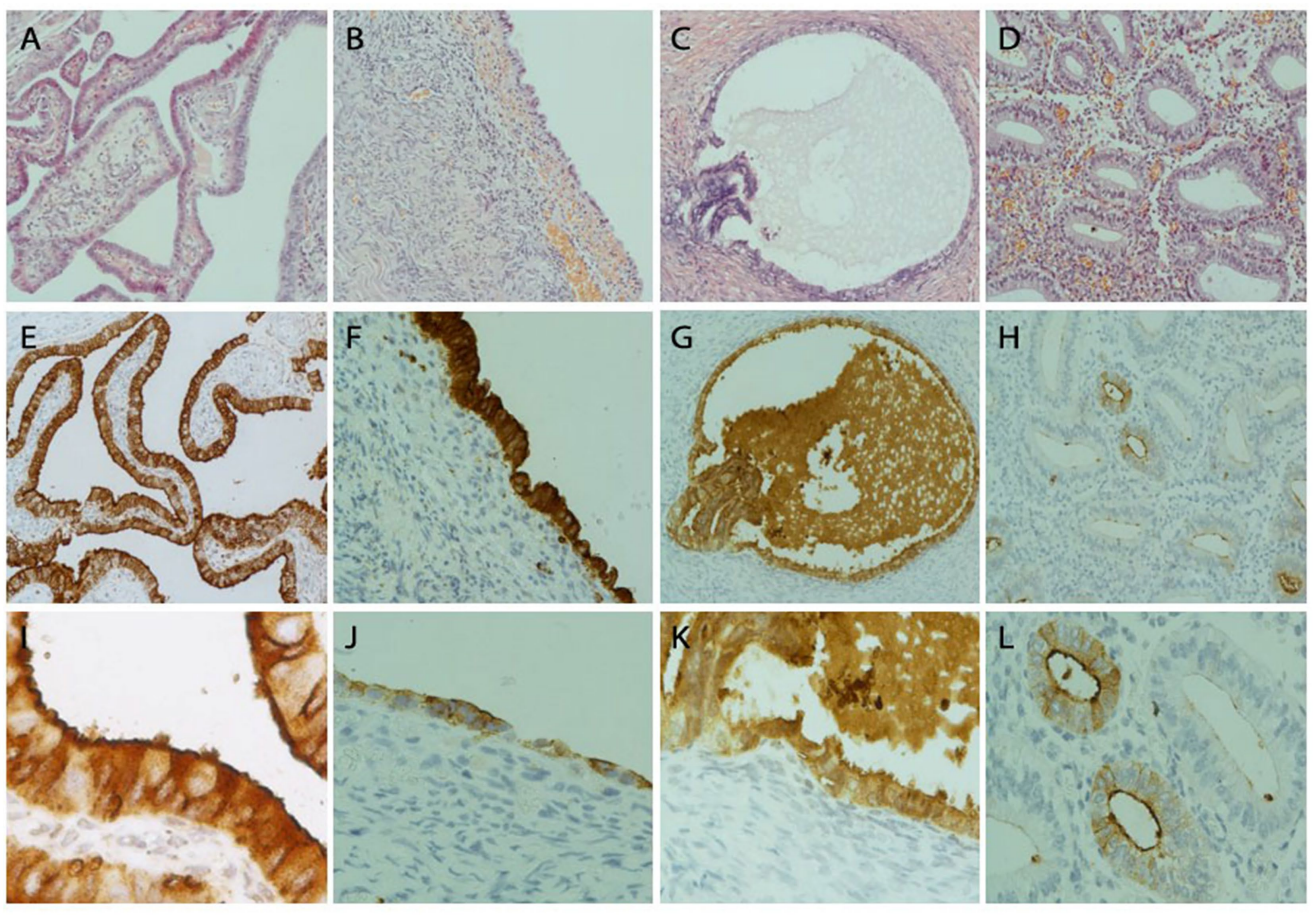
FRA protein detection by immunohistochemistry in fallopian tubal, ovarian endometriosis, endosalpingiosis, and endometrial tissue sections. Representative IHC stains of FRA showed strong diffuse FRA expression in the fallopian tube (left column, **A,E,I**), ovarian endometriosis (left mid, **B,F,J**), and ovarian endosalpingiosis (right mid, **C,G,K**), but only focal and weak staining was observed in a case of proliferative endometrium (right column, **D,H,L**). FRA staining was mainly cytoplasmic. Membranous stains can be seen when the staining intensity is moderate **(E)** and luminal stains are more prominent when stains become weak or moderate and magnified **(I,L)**. Histologic H&E sections are arranged on the top panel. FRA expression in the fallopian tube is illustrated in panels **(E,I)**, where **I** (200x) represents the magnification of part of **E** (100x). FRA expression in 2 ovarian endometriosis cases, with one showing high (**F**, 100x) and one showing intermediate (**J**, 100x) FRA expression levels. FRA expression in endosalpingiosis is shown in panels **G** (100x) and **K** (200x). Focal glandular with a mainly luminal pattern is seen in an endometrial sample (**H**, 100x; **L**, 200x).

Compared with the endometrium, both tubal and ovarian endometriosis samples showed significantly higher FRA IHC scores with *p* = 0.00051 and *p* = 0.00174, respectively. Interestingly, the difference of FRA expression between the fallopian tube and ovarian endometriosis also reached a statistical significance (*p* = 0.05) with the expression was higher in the tubal samples. The data is summarized in Figure 3, and representative IHC pictures of the FRA expression are presented in Figure 4. Stromal cells and blood vessels were all negative for FRA expression in all samples examined.

## Discussion

Endometriosis remains a serious problem for reproductive-age women. The etiology and pathogenesis of endometriosis have been the subject of numerous investigations, although answers remain unclear (4, 10, 13, 16, 17). Starting in 2005, our group described the earliest morphologic changes of endometriosis in the ovary, which we named “initial endometriosis” (4). That study, which was largely based on light microscopic observations, provided morphologic evidence that retrograde menstruation may not explain how the initial endometriosis forms either on the ovarian surface or within the ovarian cortex. That study indirectly provided some supportive evidence that ovarian endometriosis may be derived from the fallopian tube. Morphologically, foci of initial endometriosis show gradual transitions from a typical example of endosalpingiosis (tubal type epithelia with ovarian stroma but without appreciable vascular changes) to areas with a classic appearance of endometriosis (endometrioid epithelia associated with endometrioid stromal cells and increased density of microcapillary vessels) (4). Such morphologic transitions suggest that the foci of ovarian endometriosis started from the site within the ovary, rather than being deposited from retrograde menstrual endometrial tissue.

From what being presented, we questioned the hypothesis of retrograde menstruation and proposed that tubal cells are a plausible tissue source for ovarian endometriosis (18). Recent studies of the tubal origin of ovarian serous carcinomas (5, 19, 20) have highlighted many previously under-recognized biologic and physiologic properties of the fallopian tube. *In vivo*, the fallopian tube is in close spatial proximity to the ovary (5, 8, 19, 21, 22), tubal epithelia are easily sloughed from the tubal mucosae (19, 23), and the majority of the endosalpingiosis or OEIs are originated from the fallopian tube. Moreover, endosalpingiosis or OEIs are readily observed in ovarian cortex in many ovarian endometriosis (5). It is likely therefore that the relationship between endosalpingiosis and initial endometriosis represents a trans-differentiation process from the former to the latter, although detailed underlying mechanisms remain to be clarified.

The current study used another biomarker, FRA (folate receptor alpha), which is highly differentially expressed in the tubal and endometrial tissues. FRA, the product of the *FOLR1* gene, is a glycosylphosphatidylinositol (GPI)-anchored protein that binds plasma folate (5-methyltetrahydrofolate) and transports it into the cell *via* endocytosis (24). Folate is essential for 1-carbon metabolism, transferring single carbon units in reactions involving purine and pyrimidine synthesis, DNA repair, and methylation of various biomolecules including DNA, proteins, phospholipids, and neurotransmitters (25, 26). Folate deficiency has been linked with dysregulation of these processes and, in some cases, is associated with an increased risk of developing cancer, including serous type epithelial ovarian tumors (15, 27–29). However, no specific data have been reported on the expression of FRA in normal fallopian tube relative to ovarian endometriosis or to evaluate what role, if any, FRA may play in the development of ovarian endometriosis.

Since *FOLR1* and its protein product FRA were highly differentially expressed in the tube and the eutopic endometrium, we assessed the tubal and endometrial samples with the FRA antibody on 50 patients with ovarian endometriosis (32 paired and 18 non-paired). We found that all fallopian tubal epithelia expressed FRA with strong intensity. In contrast, FRA expression in the eutopic endometrium was either negative or weakly and focally positive. Therefore, FRA may be considered a tubal-specific biomarker when compared to that of the endometrium. In our previous whole-genome expression microarray analysis (10), we identified that *FMO3* was also more specific for the fallopian tube and endometriosis, but not for the endometrium, although the relationship between *FOLR1* and *FMO3* is unclear. Among 18 non-matched ovarian endometriosis, 15 cases had strong or moderately membrane and intracellular staining. Only 3 (18%) samples showed a weak staining on apical luminal borders. Furthermore, Pax-8 positive epithelial cells on the ovarian surface and OEIs showed moderate to strong FRA expression, while Pax-8 negative epithelial cells on the ovarian surface and OEIs were negative for FRA, which is supportive of tubal origin of ovarian endometriosis. Morphologically ovarian surface like epithelia and OEIs have two origins with one originated from classic OSE (Pax-8 negative) and the other from fallopian tube (Pax-8 positive) (5). Original OSE negative for FRA expression suggests that ovarian endometriosis is unlikely derived from OSE through a metaplastic process. In short, our findings from this study further support the hypothesis that ovarian endometriosis is at least partially derived from the fallopian tube. In our previous study (10), we estimated that approximately 60% of ovarian endometriosis may be originated from the tubal epithelia based on the *FMO3* and *DMBT1* expression study. This is correlated to the number of OEIs from fallopian tube (5). The design of the current study does not allow such quantification; however, the findings bolster the argument that the majority of ovarian endometriosis comes from the fallopian tube rather than from retrograde menstrual endometrium.

**Figure 5:**
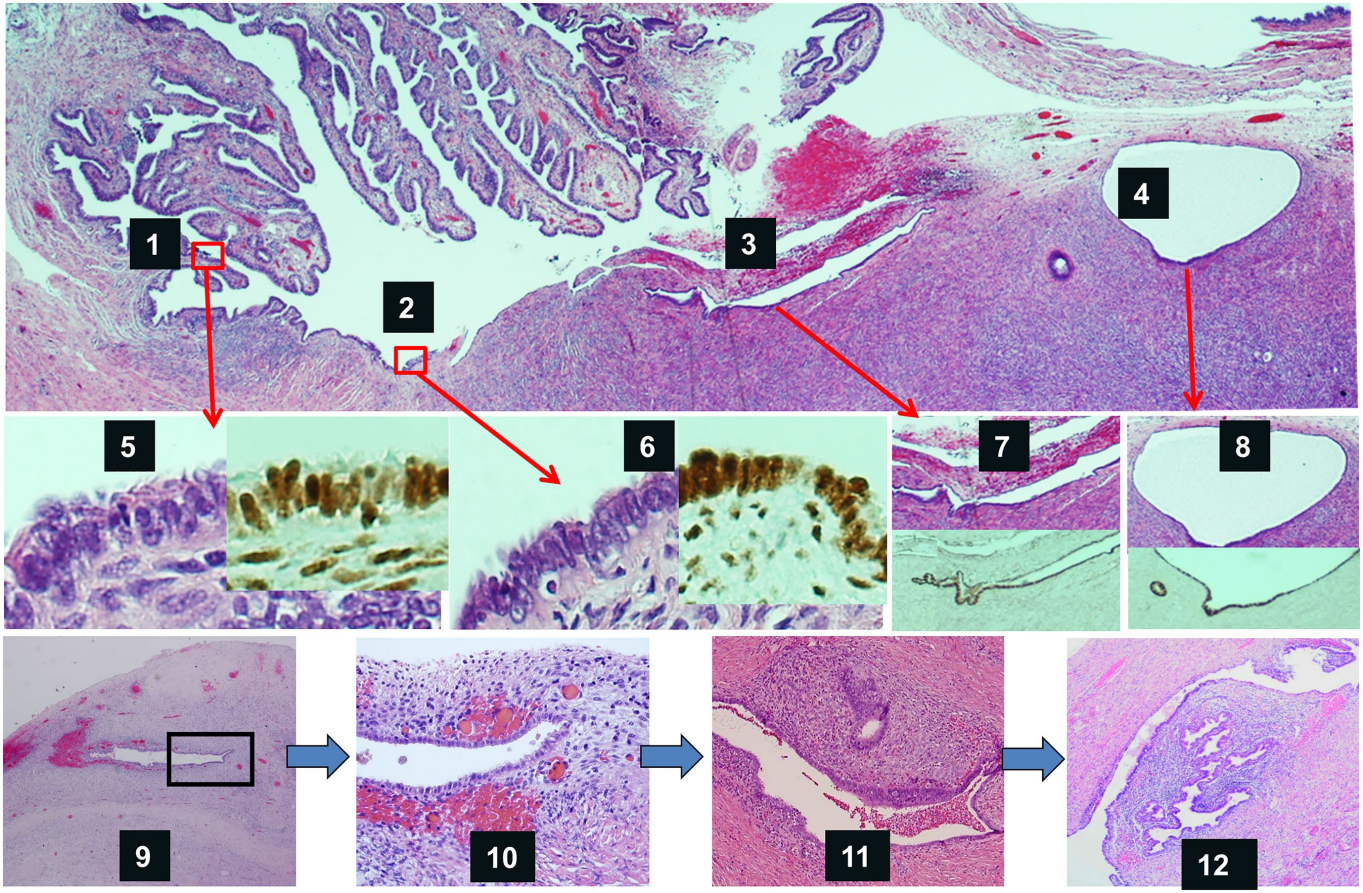
Graphic abstract of the process of ovarian endometriosis developed from tubal epithelia. Top panel shows tubal fimbriated end adherent to the ovary. Tubal epithelia (1) spreads to the ovarian surface (2), invaginates onto ovary through adhesions (3), and forms endosalpingiosis or ovarian epithelial inclusions (4). Mid-panel shows magnified morphology as well as Pax-8 IHC stains of tubal epithelia (5), epithelia on the ovarian surface (6), epithelia within ovarian surface adhesions (7), and endosalpingiosis (8). Low-panel shows endometriosis develops from initial endometriosis within the ovary. Endosalpingiosis like gland start to have endometrioid stroma and increased micro capillary vessels (9), more apparent in a magnified view (10), endometriosis without evidence of bleeding (11), and typical appearance of endometriosis in the ovary (12).

Two essential conditions must be met to consider the fallopian tube to be the origin of endometriosis when present in the ovary. First, tubal cells must enter the ovary. Frequent detachment of tubal epithelia from fimbriated ends makes this possible. Tubal cells are easily retrieved by flushing the fallopian tube (19, 23). The process is further facilitated by juxtaposed spatial relationship between tubal fimbria and ovarian surface (21, 30). The rupture of ovarian surface caused by ovulation (31, 32) provides a favorable condition for tubal epithelia to implant onto the ovary then get into ovarian cortex. The latter process, from a morphologic perspective, has long been described as endosalpingiosis (33–35). Second, endosalpingiosis or OEI must transform itself into endometriosis. The latter probably occurs through trans-differentiation, a process that is commonly seen in the Müllerian system (36), although detailed molecular mechanism remains elucidated. Initial endometriosis within the ovary describes the morphologic transition of OEIs, with some glands of OEIs displaying the earliest morphologic changes of endometriosis in only half of the gland (4). Trans-differentiation from tubal epithelia is the most likely explanation for this morphologic observation, especially since transitional areas from normal-appearing tubal epithelia to endometrial like tissue are commonly present (19, 37). In summary, a graphic abstract is illustrated in Figure 5 to aid understanding to process of endometriosis formation within the ovary. We may conclude, therefore, endometriotic or endometrioid epithelial cells are likely originated from the tubal epithelia. However, by all means, so far we do not have solid scientific evidence that ovarian endometriosis are either derived from or not coming from the endometrial cells from retrograde menstruation. Further studies in this regard are needed.

FRA came into focus recently as an anticancer target after the successful development of drugs targeting intracellular folate metabolism and protein location in the cytoplasm or on cell membrane. Binding to FRA is one of several methods by which folate is taken up by cells; however, the receptor FRA is an attractive anticancer drug target owing to the overexpression of FRA in various types of cancers, including ovarian epithelial carcinomas (38). Treatment of endometriosis is typically either hormonal therapy or surgical excisions. Interestingly to note that expression of FRA was detected in 94.4% of endometriosis samples from 18 patients in a targeted intraoperative imaging study (39) Considering FRA is highly expressed in ovarian endometriosis, targeted therapy against FRA deserves more exploratory studies. In addition, the finding of FRA highly expressed in the fallopian tubal epithelial cells may suggest an alternative way to prevent tubal derived ovarian serous carcinomas as well as possible ovarian endometriosis. The novel findings of this study have provided further evidence supporting our previously proffered theory of the tubal contributions for ovarian endometriosis. Although our findings remain preliminary, they might provide an alternative way of thinking about the etiology and pathogenesis of endometriosis that might aid the prevention and early treatment of ovarian endometriosis.

## Conclusion

The folate receptor alpha gene *FOLR1* identified through a differential gene array analysis is highly expressed in ovarian endometriosis and the fallopian tube epithelial cells. However, the expression level of *FOLR1* and its protein FRA is significantly lower in the endometrium and ovarian surface epithelia. These novel findings further support that the fallopian tube is likely the cellular source of ovarian endometriosis. Understanding the tubal contribution to ovarian endometriosis should ultimately contribute to ongoing investigative efforts aimed at identifying alternative ways to prevent and treat endometriosis.

## Materials and methods

### Tissue specimens

Fresh tissue samples, including normal fallopian tube fimbria and corresponding endometrium were obtained from pathology specimens within 30 min of their surgical resections as described previously (10). A total of 114 formalin fixed and paraffin-embedded tissues were obtained from Henan Provincial People’s Hospital, Zhengzhou, China. Among 114 samples, 96 were derived from 32 patients with each patient contributing 1 ovarian endometriosis, 1 endometrium and 1 fallopian tube sample and 18 patients each contributing one sample of ovarian endometriosis only. In all 50 ovarian endometriosis samples studied, 42 were endometriomas or endometriotic cyst, 6 were endometriosis foci within the ovarian parenchyma without cyst or mass formation, and 2 were endometriosis on the ovarian surface. Among the 32-paired samples, 15 had fresh tissue samples available in addition to the formalin-fixed paraffin sections. The remaining 18 patients had ovarian endometriosis from formalin-fixed tissues only. In addition, 30 ovarian tissue sections containing Pax-8 negative OSE were included for FRA IHC stains. The representativeness of the ovarian endometriosis foci (n = 15) for quantitative PCR and Western blot analysis were confirmed by examining the corresponding frozen sections under the microscope. Foci of endometriosis, comprised predominantly of epithelial and stromal tissue, were manually dissected by removing non-endometriotic ovarian tissue.

Patients’ age ranged from 22 to 50 years (mean 34.5). The reasons for total hysterectomy and bilateral salpingo-oophorectomy were prolonged ovarian endometriosis or benign gynecologic disorders including leiomyomata and benign ovarian cysts. No patient received hormonal treatment in 12 months prior to the surgery. Pathologic diagnosis of ovarian endometriosis were confirmed by two pathologists (RZ and WZ). Endometriosis, tubal mucosa and endometrium with both glandular epithelia and stroma were confirmed to be present under microscope. Determination of menstrual cycle phase (proliferative or secretory) for the 15-paired cases with both fresh and formalin-fixed tissues was made by examining hysterectomy specimens microscopically. Patients with a history of malignancy or tubal ligation were excluded. The research protocol was approved by the institutional review board of Henan Provincial People’s Hospital at Zhengzhou, Henan Province, China.

### Microarray and data analysis

In order to identify tissue-specific biomarkers, we compared the gene expression profiles between the fallopian tube and the endometrium from patients without evidence of endometriosis by gene array analysis (10). The endometriosis samples were manually dissected after microscopic confirmation. Three pairs of fresh samples of the fallopian tube fimbria and corresponding endometrial specimens from 3 reproductive-age women were collected and sent to Kang Chen Bio-Tech (Shanghai, China) to perform whole-genome expression microarray analysis using the Agilent array platform (Agilent Technologies, Palo Alto, CA, United States), as previously described (10). To limit potential confounding interference, endometrial samples showing tubal metaplasia were excluded from the study. Total RNA from three pairs of hand-dissected epithelial samples under routine microscopy were prepared by using TRIzol (Invitrogen, Gaithersburg, MD, United States), further quantified by the NanoDrop 1,000, and RNA integrity was confirmed by standard denaturing agarose gel electrophoresis. The Human Gene Expression Array was manufactured by Agilent with a cluster of 41,000 genes and their corresponding transcripts and they are present in those public domain annotations.

Array hybridization and sample labeling were done according to the protocol provided by Agilent Technologies (Palo Alto, CA, United States) as described elsewhere (40). Median normalization and subsequent data processing were analyzed by using the GeneSpring GX software (version 11). Highly differentially expressed genes were selected for further analysis after normalization of the raw data. Differentially expressed genes were identified through fold change filtering. Hierarchical clustering analyses were carried out also by using the Agilent GeneSpring GX software. Analysis of gene ontology and pathway studies were performed by using the standard enrichment computation method. A gene was considered to be highly differentially expressed between the tubal fimbria and the endometrium if there was a ≥2-fold difference in its expression level between or among the tissue categories, and the *p*-value less than 0.05 was considered significant.

### Validation of microarray data by real-time PCR

Multiple differentially expressed genes were verified and published previously (10). In the current study, we examined another highly differentiated gene *FOLR1* to further test the possibility of tubal contribution of ovarian endometriosis.

To verify the gene expression data obtained from the microarray, real-time PCR was performed on *FOLR1* gene, using total RNAs from 15-paired tubal fimbria and corresponding endometrial samples. Among the 15-paired specimens, 12 were in the proliferative phase and the remaining 3 were secretory endometria, which were determined based on endometrial morphology. The *FOLR1* gene was selected in this study because it was 56-fold more expressed in the tubal samples compared with the endometria by gene array study. In addition, the antibody against corresponding protein FRA suitable for immunohistochemistry on FFPE tissue samples was recently available. FRA represents one of the glycosylphosphatidylinositol (GPI)-anchored receptors that bind plasma folate (5-methyltetrahydrofolate) with high affinity (K_D_ ~ 1 nM), and transport it into the cell *via* endocytosis (24). Together with folate receptor beta, gamma, and delta, FRA lies in tandem on chromosome 11q13 (24), although much less is known about those other family members. FRA has been shown to be expressed in normal placenta, fallopian tube, kidney, lung, breast and choroid plexus (15).

GAPDH was used as internal control. Real-time PCR was done on 15-paired ovarian endometriosis, tubal, and endometrial samples. Primers were designed using Primer 3 software and the sequences were as follows.

*FOLR1:* F5′-CCCGAGGACAAGTTGCATGA-3′

R5′-TCCACAGTGGTTCCAGTTGAATCTA-3′

*GAPDH*: F5′-AGCAAGAGCACAAAGAGGAAGAG-3′

R5′-TCTACATGGCAACTGTGAGGAG-3′

The protocol of real-time PCR was described elsewhere (41). Data analysis was executed with StepOnePlus™ Real-Time PCR System software, version 2.2 (Applied Biosystem, Hercules, CA). Comparative Ct method (ΔΔCt) was used to obtain relative quantification of the gene expression levels. Quantification of the amplified products were normalized against GAPDH (ΔCt). Paired two-tailed t test was used to compare relative mRNA expression levels of the studied tissue samples. Statistical significance was defined as a *p* value < 0.05.

### Western blot analysis

Monoclonal antibodies against FRA, 1:500 dilution, was obtained from Dr. Daniel O’Shannessy (Department of Diagnostics Development, Morphotek Inc., Exton, Pennsylvania). All samples mentioned above were subsequently evaluated for protein expression by Western blot, as described elsewhere (42). GAPDH antibody (Abcam, United States) was used as the loading control.

### Immunohistochemistry

Among many differentially expressed genes, folate receptor-alpha (FRA) was one of the most highly differentially expressed. Immunohistochemistry (IHC) stain with antibodies against FRA, mentioned above, was performed as described previously (15). Normal fallopian tube samples served as a positive control since it is ubiquitously expressed in tubal epithelial cells (15). Negative controls were carried out by replacing primary antibodies with class-matched mouse and rabbit IgGs on parallel sections. The subcellular staining localization for FRA was both cytoplasmic and membranous. Rabbit monoclonal anti-PAX8 antibodies were purchased from Abcam (Boston, United States). Positive and negative controls for PAX8 IHC were the same as anti-RFA.

IHC stained slides were evaluated and scored by counting at least 500 epithelial cells independently by two pathologists (RZ and WZ). Positive FRA expression was defined as discrete membrane and intracellular (mainly cytoplasmic) brown color with at least weak intensity within the epithelial cells. IHC scoring criteria have been described previously (15). Briefly, the staining intensity was scored as 0 if negative, 1 if with weak intensity, 2 with moderate intensity, and 3 if strong and intensely stained. Tissues with score 3 staining showed an intensely positive pattern that was readily identifiable at low magnification (4× objective) under light microscope. Sometimes a complete circumferential staining was seen. Score 2 was only visible at the 10× objective level and it was typically localized to the apical luminal or occasionally to the lateral cell border. In contrast, score 1 stain was generally limited to the luminal borders, or required 20× to 40× objectives to confirm. Percentage of the positive cells at each intensity was calculated for each case. The final score was summarized by multiplying the staining intensity and the percentage of the positive cells for each sample (10).

### Statistical analysis

Multiple comparisons among categories of ovarian endometriosis, benign fallopian tube and endometrial tissues were carried out by using Mann–Whitney U test and SPSS statistical software program version 13.0 (SPSS, Chicago, IL, United States) when appropriate. *p* value < 0.05 was considered statistically significant.

## Data availability statement

The raw data supporting the conclusions of this article will be made available by the authors, without undue reservation.

## Ethics statement

The research protocol was approved by the institutional review board of Henan Provincial People’s Hospital at Zhengzhou, Henan Province, China. The current study does not require patient consent to take part in this study since only residual tissues in the paraffin blocks were used after clinical diagnosis and management were completed.

## Author contributions

WZ, YiW, and YuW conceived the study design and experiments. QL, YuW, RZ, ZY, JZ, and YaW carried out experiments and data analysis. QL and WL performed data analysis. QL, YiW, WZ, RZ, and OF wrote the manuscript. YiW, YuW, RZ, and WL provided the majority of cases with relevant clinical information. All authors contributed to the article and approved the submitted version.

## Funding

This work was partially supported by grants from the National Natural Science Foundation of China (project numbers: 81972441 and 82072883). The project was also supported in part by Mark and Jane Gibson Endowment fund to WZ.

## Conflict of interest

The authors declare that the research was conducted in the absence of any commercial or financial relationships that could be construed as a potential conflict of interest.

## Publisher’s note

All claims expressed in this article are solely those of the authors and do not necessarily represent those of their affiliated organizations, or those of the publisher, the editors and the reviewers. Any product that may be evaluated in this article, or claim that may be made by its manufacturer, is not guaranteed or endorsed by the publisher.

## Abbreviations

FOLR1: Folate receptor alpha gene
FRA: Folate receptor alpha protein
GPI: Glycosylphosphatidylinositol
IHC: Immunohistochemistry
OEI: Ovarian epithelial inclusions
OSE: Ovarian surface epithelia
